# Planetary Gears Feature Extraction and Fault Diagnosis Method Based on VMD and CNN

**DOI:** 10.3390/s18051523

**Published:** 2018-05-11

**Authors:** Chang Liu, Gang Cheng, Xihui Chen, Yusong Pang

**Affiliations:** 1 School of Mechatronic Engineering, China University of Mining and Technology, Xuzhou 221116, China; jsxzlc@foxmail.com; 2 College of Mechanical and Electrical Engineering, Hohai University, Changzhou 213022, China; chenxh@hhu.edu.cn; 3 Faculty Mechanical, Maritime and Materials Engineering, Delft University of Technology, Delft 2628 CD, The Netherlands; Y.Pang@tudelft.nl

**Keywords:** planetary gear, partition, feature extraction, degradation, VMD, SVD, CNN

## Abstract

Given local weak feature information, a novel feature extraction and fault diagnosis method for planetary gears based on variational mode decomposition (VMD), singular value decomposition (SVD), and convolutional neural network (CNN) is proposed. VMD was used to decompose the original vibration signal to mode components. The mode matrix was partitioned into a number of submatrices and local feature information contained in each submatrix was extracted as a singular value vector using SVD. The singular value vector matrix corresponding to the current fault state was constructed according to the location of each submatrix. Finally, by training a CNN using singular value vector matrices as inputs, planetary gear fault state identification and classification was achieved. The experimental results confirm that the proposed method can successfully extract local weak feature information and accurately identify different faults. The singular value vector matrices of different fault states have a distinct difference in element size and waveform. The VMD-based partition extraction method is better than ensemble empirical mode decomposition (EEMD), resulting in a higher CNN total recognition rate of 100% with fewer training times (14 times). Further analysis demonstrated that the method can also be applied to the degradation recognition of planetary gears. Thus, the proposed method is an effective feature extraction and fault diagnosis technique for planetary gears.

## 1. Introduction

Because of their small size, reduced weight, and large transmission ratio advantages, planetary gear transmissions are widely used in large-scale complex mechanical systems under low speed and heavy load conditions [1]. A planetary gear transmission is more complex compared with a gear transmission with fixed axes, and its vibration signal has more intense nonlinear and nonstationary characteristics due to the influences of different working conditions, different errors, transmission paths, and other factors [2,3]. Furthermore, heavy load machinery typically includes poor working conditions that cause strong noise interference in the process of vibration signal acquisition. These factors cause fault feature information to be obscured by noise during early stage planetary gear faults and increase the difficulty of fault feature extraction. The neglected faults continue to deteriorate and affect equipment operation safety. Therefore, studying a fault diagnosis method that can effectively extract and identify weak fault feature information of planetary gears is necessary.

The collected vibration signal typically contains a significant amount of interference information due to ambient noise and the complexity of the transmission system. In particular, early fault features are weak and basically submerged in noise [4]. Directly diagnosing the original signal is difficult, and decomposition is required. Some classical mode decomposition methods based on recursion, such as empirical mode decomposition (EMD) or local mean decomposition (LMD), have been widely used in the fields of signal decomposition and feature extraction. EMD is a time-frequency analysis method used to adaptively decompose a nonlinear and nonstationary vibration signal into several strictly defined intrinsic mode functions (IMFs) [5,6]. However, end effect and modal aliasing are frequently observed in the EMD process, and the rigorous mathematical theory basis is insufficient [7]. Although these problems are mitigated by subsequent improvements or new methods, they still exist. To add white noise to improve performance, hundreds of EMD or LMD operations must be repeated and the efficiency is reduced [8,9]. In addition, false components may appear. Variational mode decomposition (VMD) is a non-recursive signal decomposition method proposed by Dragomiretskiy et al. [10]. By iteratively searching the optimal solution of variational model, the signal components are automatically decomposed in Fourier domain. Finally, modes and corresponding center frequencies are extracted [11,12].

Feature extraction is an important part of mechanical fault diagnosis [13]. Its goal is to extract low-dimensional data that contains fault feature information from high-dimensional data. Although components of the original signal become clear after decomposition, each mode is nonstationary and its fault feature information remains weak [14,15]. Signal dimensions increase and simple analysis is insufficient; hence, further extraction is required. Singular value decomposition (SVD) is a method of matrix orthogonal decomposition. It can effectively reflect the features of a matrix because singular values are matrix intrinsic characteristics. SVD has been widely used in the field of fault detection and diagnosis because of its remarkable benefits in signal denoising and feature extraction under complex noise conditions [16,17]. Zhang et al. [18] solved the problem of introducing a tight frame constraint into the popular dictionary learning model using a hard thresholding operation and SVD. Hence, a novel multiple feature recognition framework was established. Feng et al. [19] exploited the highly flexible and adaptive characteristic of the shift-invariant K-means singular value decomposition (SI-K-SVD) dictionary learning method to extract the latent components of complex signals and suppress background noise. When using SVD to process gear vibration signals, the general approach involves constructing a Hankel matrix of the original signal or IMF containing main fault feature information, and then obtaining singular values by SVD to extract information or reconstruct the signal without noise [20,21]. In this process, weak fault feature information can be eliminated or lost, which can influence the accuracy of subsequent diagnosis. Therefore, determining a more comprehensive method of extraction and identification for early weak faults in strong noise is necessary. The idea of dividing and intercepting a part of data for local processing has been applied in many fields, including when considering planetary gear vibration signals. In this study, a method of extracting local fault feature information and then composing global feature is introduced. Different gear fault states are described by the distribution and variation in local features and detailed extraction of planetary gear vibration signals is realized.

After obtaining gear fault feature information using the partition feature extraction method, effective fault identification is required to achieve an accurate diagnosis. Many traditional classifiers, such as support vector machine (SVM) and back propagation (BP) neural networks, have been used by researchers [22]. A variety of improved or new algorithms have been proposed or applied to the field of fault identification and classification, and have greatly improved the diagnostic accuracy [23,24]. However, many of these methods continue to experience difficulties when addressing large or multidimensional data samples [25], or use fewer feature parameters as input samples, which is not conducive to accurate identification. Therefore, more efficient algorithms are still needed. Convolutional neural network (CNN) is an important depth-learning model that resolves the bottleneck of traditional neural networks [26]. The structure model of a cat’s visual system was referenced during the CNN design process. Weight sharing is adopted to reduce computational complexity in the learning process and spatial correlation of the data is extracted using a local sensing area to significantly reduce the network parameters. Therefore, CNN is especially suitable for processing images or other multidimensional data, having high robustness to a certain degree of translation, scaling, and distortion [27]. Chen et al. [28] used two stacked CNNs to build a novel deep image saliency computing framework. The proposed framework highlights the objects of interest from complex background while preserving details. Levine et al. [29] developed a method based on a partially observed guided policy search method and CNN, which was used to learn policies that map raw image observations directly to torques on the robot's motors. Google DeepMind used CNN as one of the core algorithms for AlphaGo [30] and consecutively defeated several top chess players in the world, which demonstrates artificial intelligence transcending that of humans in the Go field. CNN can be used to directly process large data or multidimensional data samples, which is beneficial for more detailed local feature extraction and retaining the relative relationship of multidimensional data, leading to the acquisition of improved identification results.

In this paper, a novel method for fault feature extraction and diagnosis of planetary gears is proposed. Based on VMD and SVD, feature information is extracted in detail by partition processing, and fault classification is achieved using CNN. The remainder of this paper is composed as follows: In Section 2, the mathematical model of partition fault feature information extraction of planetary gears based on VMD and SVD is established. In Section 3, under different fault conditions, the vibration signal of a two-stage planetary gear of the drivetrain dynamics simulator (DDS) bench is measured using vibration acceleration sensors. In Section 4, the collected vibration signal is decomposed by VMD and the obtained IMFs are constructed into IMF matrices. The partition scales are defined and each IMF matrix is divided to submatrices. The singular value vectors of the submatrices are obtained using SVD and singular value vector matrices of the corresponding fault states are constructed. Finally, the singular value vector matrices containing main fault feature information are used as inputs to train and test the CNN. Then, the identification and classification of planetary gear faults is achieved. The experimental results confirm that the fault state of the planetary gears can be accurately distinguished using the proposed method. In Section 5, the performed gear degradation experiment is outlined. Different degradation states were identified effectively, and the effectiveness and the potential of proposed method are further verified. In the last section, conclusions are summarized.

## 2. Model Establishment

The mathematical model for this novel fault feature extraction and diagnosis method of planetary gears was established, which includes two parts. In the first part, signal modes were obtained using VMD to decompose the original vibration signal. Then, a method of extracting local fault feature information and composing global feature was adopted. Data compression and feature extraction were completed by dividing the mode matrix into submatrices using SVD. In the second part, CNN was used to perform feature identification. Finally, accurate fault classification and diagnosis was achieved.

### 2.1. Partition Fault Feature Extraction Based on VMD and SVD

#### 2.1.1. Variational Mode Decomposition

VMD is a new signal decomposition method based on Wiener filtering, Hilbert transform, and frequency mixing that automatically decomposes the input signal *f* into *K* modes with sparse characteristics. Each mode is assumed to have a limited bandwidth with a corresponding center frequency. By minimizing the sum of the estimated bandwidth of the modes, VMD constructs the mode decomposition problem as a process of solving for the optimal solution of a constrained variational problem.

The analytic signal $z_{k}(t)$ and unilateral frequency spectrum of mode $u_{k}(t)$ can be obtained by Hilbert transform. Then $z_{k}(t)$ is mixed with estimated center frequency $\omega_{k}$ to move the mode’s frequency spectrum to baseband. Finally, the squared *L2*-norm of the gradient is calculated to estimate bandwidth, and the constrained variational problem is constructed as follows:

(1) $$\left\{ \begin{array}{l} \min_{\left\{u_{k}\right\},\left\{\omega_{k}\right\}}\left\{\sum_{k}{\left\|\partial_{t}z(t)e^{-j\omega_{k}t}\right\|}_{2}^{2}\right\}\\ s.t.\;\sum_{k}u_{k}=f(t) \end{array}\right.$$

where the analytic signal $z_{k}(t)=[\delta (t)+j/\pi t]*u_{k}(t)$, *k* = 1, 2, …, *K*.

To unconstrain the problem, the quadratic penalty term *α* and Lagrangian multiplier λ are introduced. They can simultaneously ensure both the reconstruction precision and the constraint strictness in the presence of Gaussian noise. Thus, the augmented Lagrangian is obtained as follows:

(2) $$L\left(\left\{u_{k}\right\},\left\{\omega_{k}\right\},\lambda \right)=\alpha \sum_{k}{\left\|\partial_{t}z(t)e^{-j\omega_{k}t}\right\|}_{2}^{2}+{\left\|f(t)-\sum_{k}u_{k}(t)\right\|}_{2}^{2}+\langle \lambda (t),f(t)-\sum_{k}u_{k}(t)\rangle$$

Initialize $u_{k}$, $\omega_{k}$, and *λ*. The constrained variational problem is solved using the alternate direction method of multipliers (ADMM) to iteratively update and find the saddle point of the augmented Lagrangian. The (*n* + 1)th iterative results in the Fourier domain are easily obtained as:

(3) $${\hat{u}}_{k}^{n+1}(\omega )=\frac{\hat{f}(\omega )-\sum_{i\neq k}{\hat{u}}_{i}(\omega )+\frac{\hat{\lambda }(\omega )}{2}}{1+2\alpha {\left(\omega -\omega_{k}\right)}^{2}},$$

(4) $$\omega_{k}^{n+1}=\frac{\int_{0}^{\infty }\omega {\left|{\hat{u}}_{k}(\omega )\right|}^{2}d\omega }{\int_{0}^{\infty }{\left|{\hat{u}}_{k}(\omega )\right|}^{2}d\omega }$$

(5) $${\hat{\lambda }}^{n+1}(\omega )={\hat{\lambda }}^{n}(\omega )+\tau \left[\hat{f}(\omega )-\sum_{k}{\hat{u}}_{k}^{n+1}(\omega )\right],$$

Continue the iteration until $\sum_{k}{\left\|u_{k}^{n+1}-u_{k}^{n}\right\|}_{2}^{2}/{\left\|u_{k}^{n}\right\|}_{2}^{2}<\varepsilon$. After the end of the iteration, the time domain mode $u_{k}(t)$ is obtained using the real part of the inverse Fourier transform.

#### 2.1.2. Singular Value Decomposition

SVD is an effective matrix analysis tool that can be used to decompose and transform a matrix. Because of its unique advantages, SVD has been widely used in noise reduction, data compression, and feature extraction. An *m* × *n* matrix ***X*** consisting of original data can be represented as follows:

(6) $$X=\left[\begin{array}{cccc} x_{11} & x_{12} & ... & x_{1n}\\ x_{21} & x_{22} & ... & x_{2n}\\ ... & ... & ... & ...\\ x_{m1} & x_{m2} & ... & x_{mn} \end{array}\right]\in R^{m\times n}.$$

The existence of two orthogonal matrices $U\in R^{m\times m}$ and $V\in R^{n\times n}$ allow matrix ***X*** to be represented as:

(7) $$X=USV^{T}.$$

This equation is the singular value decomposition of matrix ***X***. Matrix ***U*** is the left singular matrix; matrix ***V*** is the right singular matrix. Matrix ***S*** is the singular value matrix, which can be represented as:

(8) $$S=\left[\begin{array}{cc} S_{1} & O\\ O & O \end{array}\right]\in R^{m\times n},$$

(9) $$S_{1}=\mathrm{diag}(\sigma_{1},\sigma_{2},\ldots ,\sigma_{r}),$$

where $\sigma_{1}\geq \sigma_{2}\geq \ldots \geq \sigma_{r}>0=\sigma_{r+1}=\sigma_{r+2}=\ldots =\sigma_{d}$, *r* = rank(*X*), *d* = min(*m*, *n*), and $\sigma_{i}$(*i* = 1, 2, …, *d*) is called the singular value of matrix ***X***. The ***O***s represent the submatrices containing the zero elements in ***S***, and their sizes depend on the relationship between *r*, *m,* and *n*.

Equation (7) can be represented as follows using column vectors $u_{i}$ of ***U*** and column vectors $v_{i}$ of ***V***:

(10) $$X=\sum_{i=1}^{d}u_{i}\sigma_{i}v_{i}{}^{T}.$$

The above equations indicate that each singular value includes different information that can reflect intrinsic characteristics of matrix ***X***. The singular value can be used as the feature parameters after compression and feature extraction of the vibration signals.

#### 2.1.3. Partition Fault Feature Extraction

The traditional feature extraction method frequently requires complex computation that can easily disregard weak local information when directly addressing these types of data. Sometimes, sampling frequency can be increased to improve the accuracy when collecting vibration signals of planetary gears. Moreover, the appropriate number of modes obtained by VMD can also be large. These issues introduce problems of high dimensionality and large amounts of data to feature extraction. The local fault feature information can be scattered among several IMFs or time intervals, which can be easily overlooked if directly extracting the global feature or the information of a single mode. The concept of performing local operation after partitioning data can be used to solve this problem. It is one of the important methods for data processing that is usually used in situations where detailed local information must be retained or large amounts of multidimensional data must be processes, such as images. Combined with VMD and SVD, a method for local extraction and global composition can be used to obtain the matrix data containing fault feature information as feature parameters. The feature parameters can accurately reflect the current fault state and be used to classify planetary gear faults.

The partition fault feature extraction process is as follows:

Step 1. The original vibration signal $X=\begin{array}{cccc} \left[X(1\right) & X(2) & \ldots & X(N) \end{array}]$ is decomposed by VMD and *K* modes are obtained. A *K* × *N* matrix ***A*** is constructed using mode $u_{k}(t)$ (*k* = 1, 2, …, *K*) as rows according to their decomposing sequences:
  (11) $$A=\left[\begin{array}{c} u_{1}(t)\\ u_{2}(t)\\ ...\\ u_{K}(t) \end{array}\right]=\left[\begin{array}{cccc} u_{1}(1) & u_{1}(2) & ... & u_{1}(N)\\ u_{2}(1) & u_{2}(2) & ... & u_{2}(N)\\ ... & ... & ... & ...\\ u_{K}(1) & u_{K}(2) & ... & u_{K}(N) \end{array}\right]\in R^{K\times N}.$$
Step 2. The obtained *K* × *N* matrix ***A*** contains fault feature information about the planetary gear. The partitioning method is used to extract the local fault feature information and to divide matrix ***A*** into a number of *m* × *n* submatrices (*m* < *K*, *n* < *N*). Notably, in the process of partitioning matrix ***A***, the step length for partitioning the rows is *a* and the step length for partitioning the columns is *b* (*a* ≤ *m*, *b* ≤ *n*). The extracted number of *ij* submatrices $B_{ij}$ from matrix *A* is as follows:
  (12) $$B_{ij}=\left[\begin{array}{cccc} u_{c+1}(d+1) & u_{c+1}(d+2) & ... & u_{c+1}(d+n)\\ u_{c+2}(d+1) & u_{c+2}(d+2) & ... & u_{c+2}(d+n)\\ ... & ... & ... & ...\\ u_{c+m}(d+1) & u_{c+m}(d+2) & ... & u_{c+m}(d+n) \end{array}\right]\in R^{m\times n},$$
  where i=1, 2, … ,I, j=1, 2, … ,J, I=[(K−m)/a+1], J=[(N−n)/b+1], c=a(i−1), and d=b(j−1). Overlaps exist between adjacent submatrices to consider their relationships. The degree of overlap is determined by the size of step lengths *a* and *b* relative to the submatrix parameters *m* and *n*.
Step 3. Each submatrix $B_{ij}$ is decomposed using SVD and the obtained singular value is defined as local feature parameters of matrix ***A*** in the position of submatrix $B_{ij}$. Submatrices typically satisfy *m* < *n*. Furthermore, the number of singular values of a matrix is equal to the smaller value of its rows and columns. This means that the singular value vector $S_{ij}$ of submatrix $B_{ij}$ can be obtained as follows:
  (13) $$S_{ij}={\left[\begin{array}{cccc} \sigma_{ij}(1) & \sigma_{ij}(2) & \ldots & \sigma_{ij}(m) \end{array}\right]}^{T}.$$
Step 4. The singular value vector $S_{ij}$ is arranged according to the position of submatrix $B_{ij}$ in matrix ***A*** and the singular value vector matrix ***S*** corresponding to current fault state is obtained as follows:
  (14) $$S=\left[\begin{array}{cccc} S_{11} & S_{12} & ... & S_{1J}\\ S_{21} & S_{22} & ... & S_{2J}\\ ... & ... & ... & ...\\ S_{I1} & S_{I2} & ... & S_{IJ} \end{array}\right]\in R^{mI\times J}.$$
Step 5. CNN is used to address matrix data and singular value vector matrix ***S*** is used as the input to achieve the effective identification of the fault state.

### 2.2. Convolutional Neural Network

CNN is a kind of multilayer artificial neural network with a special network structure. Its weight sharing can significantly reduce the complexity of network computing, and it can also directly process multidimensional data. Therefore, CNN has excellent advantages in solving image processing or multi-sensor signal processing problems.

**Figure 1:**
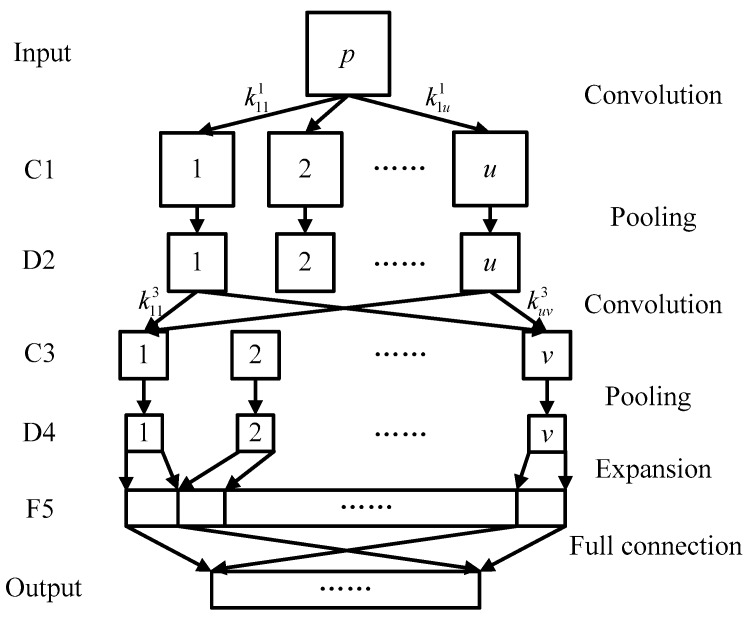
Two-convolution-layer convolution neural network (CNN).

A typical two-convolution-layer CNN structure is displayed in Figure 1. It consists of an input layer, several alternate convolution layers and down sampling layers, a feature vector layer, and an output layer. The matrix to be processed is used as the input layer for the network. It is processed with convolution operations with a number of learnable convolution kernels in the first convolution layer. The other convolution layers use the output matrices of the upper layers as inputs. The convolution results are processed by an activation function and the feature matrices of the current convolution layer are the output. The convolution equation of *M* × *N* matrix ***p***(*x*, *y*) and convolution kernel ***k***(*x*, *y*) is as follows:

(15) $$P(m,n)=p(x,y)*k(x,y)=\sum_{x=1}^{a}\sum_{y=1}^{b}k(x,y)p(m+x-1,n+y-1),$$

where the size of convolution kernel ***k***(*x*, *y*) is *a* × *b* and matrix ***P***(*m*, *n*) is the result of convolution operation that satisfies 1≤m≤(M−a+1) and 1≤n≤(N−b+1).

The activation function is typically a sigmoid function:

(16) $$f(x)=\frac{1}{1-e^{-1}}.$$

The calculation process of the convolution layer can be expressed as follows:

(17) $$p_{j}^{l}=f\left(\sum_{i\in M_{j}}(p_{i}^{l-1}*k_{ij}^{l})+B_{j}^{l}\right),$$

where $p_{j}^{l}$ is the *j*th output feature matrix of current convolutional layer *l*, $M_{j}$ is a set of input feature matrix $p_{i}^{l-1}$ corresponding matrix $p_{j}^{l}$, and $B_{j}^{l}$ is a bias corresponding to $p_{j}^{l}$.

Feature information can be fully extracted by multiple feature matrices; however, the amount of data is excessive. The down sampling layer is used to pool the output feature matrices of the convolution layer to reduce the data dimension and prevent overfitting. The calculation process of pooling an *S* × *T* matrix ***q*** is as follows:

(18) $$Q(s,t)=\frac{1}{cd}\sum_{i=1}^{c}\sum_{j=1}^{d}q(cs+i-c,dt+j-d),$$

where 1≤s≤S/c, 1≤t≤T/d, and the size of pooling area is *c* × *d*. To avoid losing excessive feature information, the size of the pooling area is not suitable if set overly large.

The convolution layers and down sampling layers are alternately arranged. Different network structures can be obtained by setting different numbers of layers and parameters. The elements of all the output feature matrices of the upper layer are expanded in sequence at the end of the neural network. These elements are used to form a feature vector that fully connects to the output layer to obtain the output of the entire network.

## 3. Test Equipment and Data Acquisition

**Figure 2:**
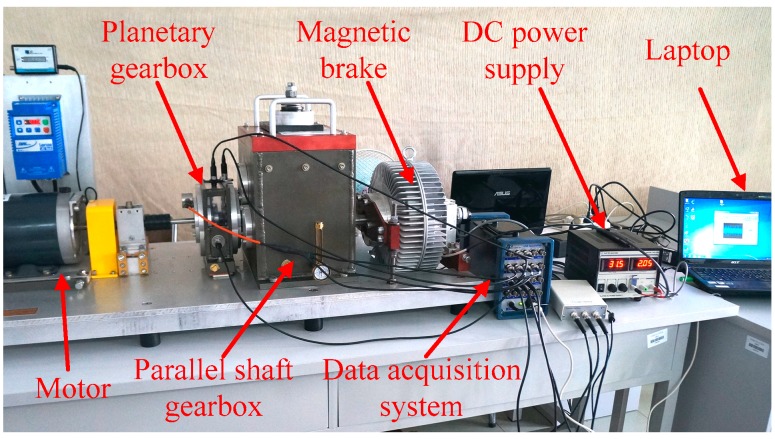
Drivetrain dynamics simulator (DDS) mechanical fault comprehensive simulation bench.

**Figure 3:**
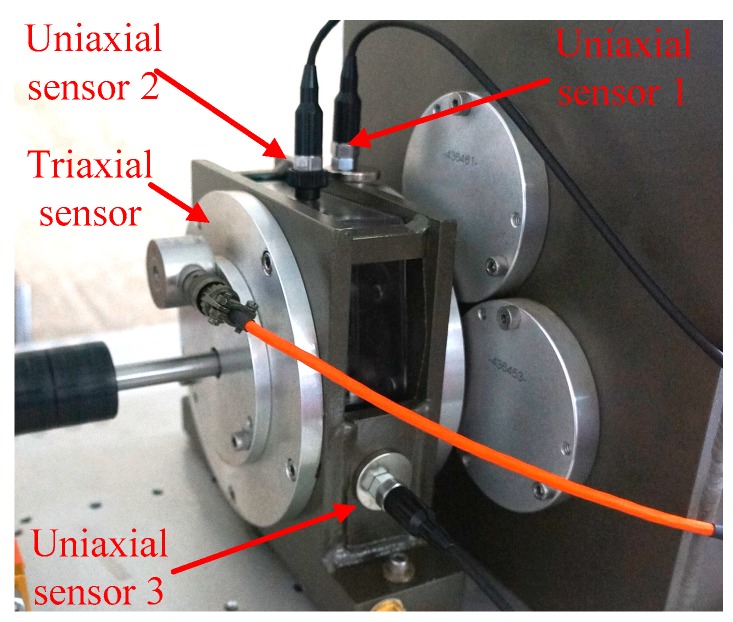
Arrangement of test points and vibration acceleration sensors.

**Figure 4:**
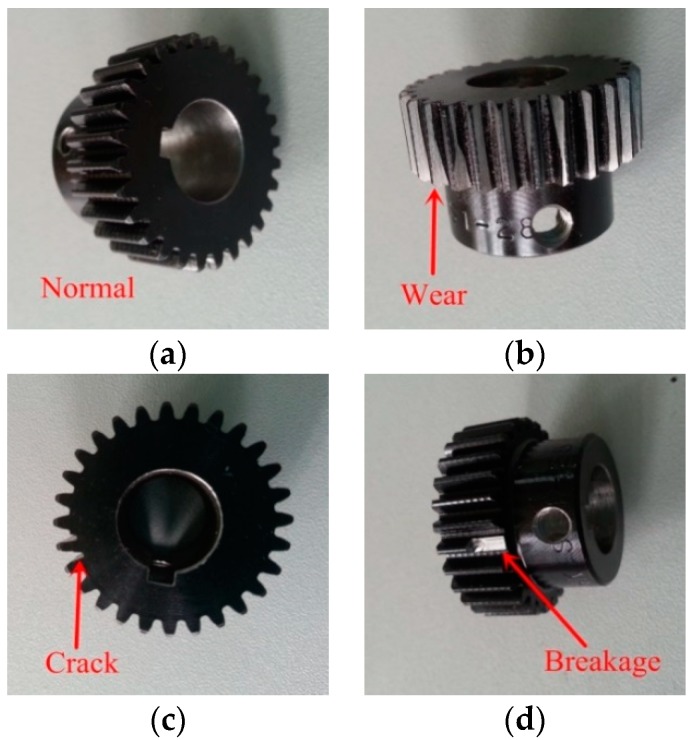
Second-stage sun gears in four states: (**a**) normal gear, (**b**) gear with wear, (**c**) gear with breakage, and (**d**) gear with root crack tooth.

The original vibration signals of the planetary gears were collected on a DDS mechanical fault comprehensive simulation bench made by Spectra Quest Company from the United States. The simulation bench displayed in Figure 2 is primarily composed of a variable-speed motor, a two-stage planetary gearbox, a two-stage parallel shaft gearbox, a magnetic brake, and other devices and sensors. The motor output frequency was 40 Hz and the magnetic brake load was adjusted to 13.5 N·m using PC-control software matched with the simulation bench. As the frequent mesh of the sun gear occurs during planetary gear transmission, faults tend to occur here. In this study, the sun gear faults of second-stage planetary gear were chosen to be studied, and the vibration signals were collected from normal gear, gear with wear, gear with root crack tooth, and gear with breakage. The arrangement of test points and vibration acceleration sensors is displayed in Figure 3 and the measured gears are displayed in Figure 4. A triaxial sensor and several uniaxial sensors were used to collect vibration signals. The sampling frequency of vibration acceleration sensors was 13 kHz. According to the effect of signal collection, the signal of uniaxial sensor 1, close to the second stage planetary gear, was selected for analysis.

## 4. Experimental Analysis

**Figure 5:**
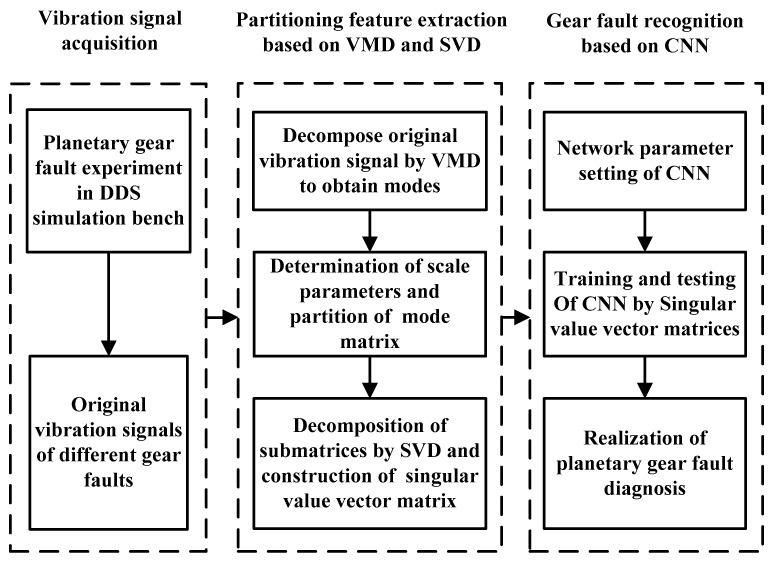
Experimental analysis flowchart.

**Figure 6:**
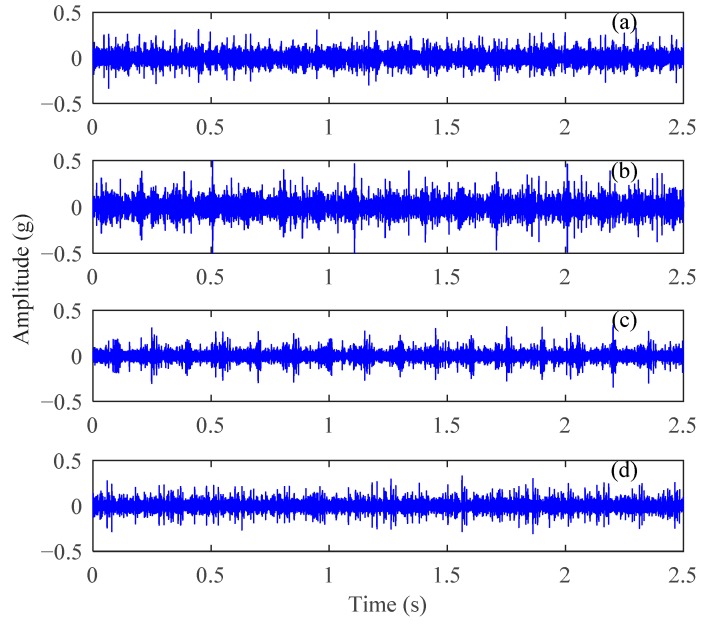
Time-domain vibration signals of the planetary gears: (**a**) normal gear, (**b**) gear with wear, (**c**) gear with breakage, and (**d**) gear with root crack tooth.

The experimental analysis flowchart of the proposed method for fault feature extraction and diagnosis of planetary gears is displayed in Figure 5. The time-domain waveforms of the vibration signals for normal gear, gear with wear, gear with root crack tooth, and gear with breakage from uniaxial sensor 1 are displayed in Figure 6. The basic parameters of the two-stage planetary gears of the DDS mechanical fault comprehensive simulation bench are presented in Table 1.

In addition to normal components, such as meshing frequencies, from the vibration signals of planetary gears in Figure 6, periodic shock components occur in the normal gear vibration signal. The periodic impact components of fault gear signals are more pronounced than those of normal gear signals. Although some differences exist between the time-domain signal waveforms of different fault state, explicitly describing them remains difficult. The frequencies of these impact components are low and inconsistent with the fault feature frequencies of the planetary gears listed in Table 1. The gear fault feature information is obscured in the time-domain waveforms of vibration signals and the gear fault state cannot be distinguished effectively.

**Figure 7:**
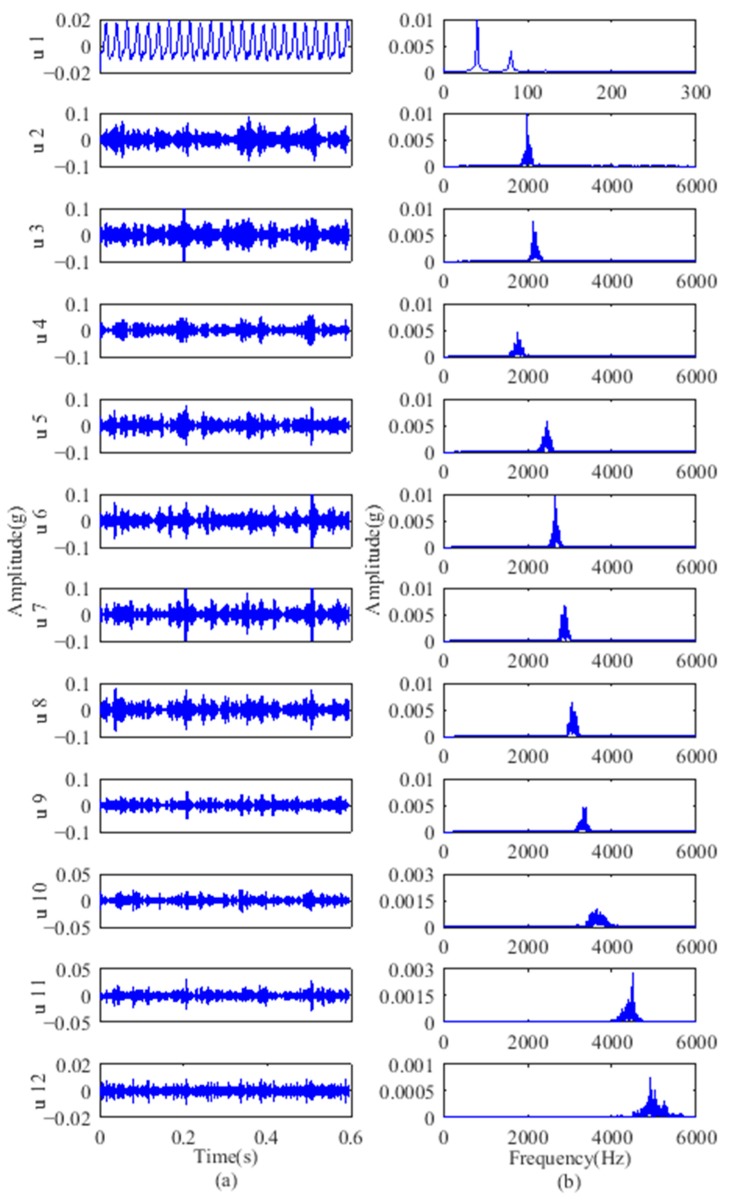
VMD results of gear with breakage: (**a**) modes; (**b**) frequency spectrums.

**Figure 8:**
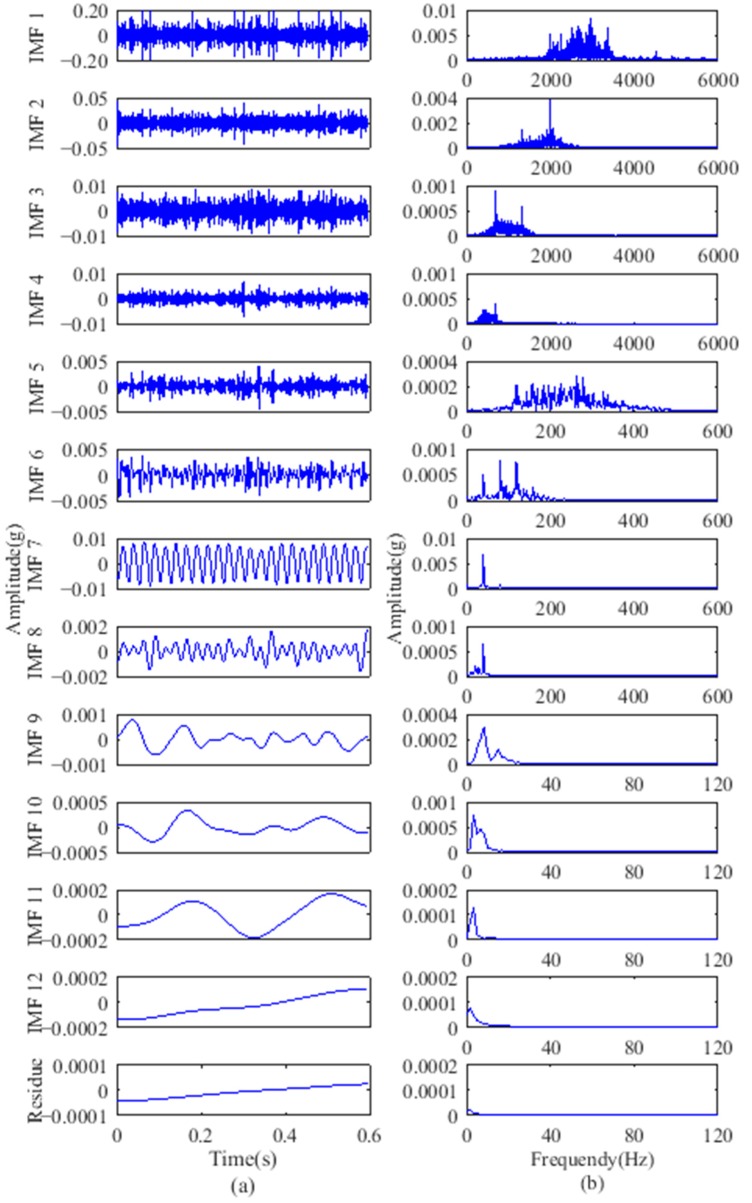
EEMD results of gear with breakage: (**a**) IMFs; (**b**) frequency spectrums.

The gear fault feature information can be extracted using the proposed method of partition fault feature extraction based on VMD and SVD. The vibration signals of the second stage sun gear fault are decomposed by VMD. The number of signal sampling points was 7808, and the corresponding time length was 0.6 s. According to the test specific conditions, while avoiding excessive decomposition, set the modes number *K* = 12, quadratic penalty term *α* = 2000, and time constant *τ* = 0. To verify the effectiveness of the proposed method, an ensemble empirical mode decomposition (EEMD) was used to compare with its results with VMD. For EEMD, the amplitude of the added white noise was set to 0.2 times the standard deviation of the original vibration signals and the number of added white noise was set to 100. As an example, the vibration signal of gears with breakage was decomposed to 12 modes using VMD, whereas 12 IMFs and a residual component are obtained using EEMD. Because of the limited space, the first to sixth modes, IMF5 to IMF10, and their spectrums are displayed in Figure 7 and Figure 8, respectively.

From Table 1, the sun gear fault feature frequency of the second stage planetary gear was 20.83 Hz. As indicated, the VMD spectrums in Figure 7 primarily intercept the 0–3000 Hz frequency band. The motor output frequency of 40 Hz and its harmonic components at 80 Hz can be observed in the first mode. The 2nd–12th modes contain primarily mid- and high-frequency components including a large amount of background noise, meshing frequencies, and their harmonic components. The EEMD spectra show that IMF1–IMF5 contain primarily high-frequency components. The motor output frequency and its harmonic components are divided into different IMFs in Figure 8. Although VMD has a good decomposition effect, the fault feature frequency components are not pronounced in either the VMD or EEMD results, and their harmonic components cannot be distinguished due to the large number of uncorrelated components in the high frequency band.

From the above analysis, the additional components do not allow obscured fault feature information to be directly distinguished from the modes. Furthermore, EEMD still includes a certain modal aliasing that can identify elusive IMFs. This means that simple time-frequency analysis for IMFs is insufficient and further extraction on this basis is required.

**Figure 9:**
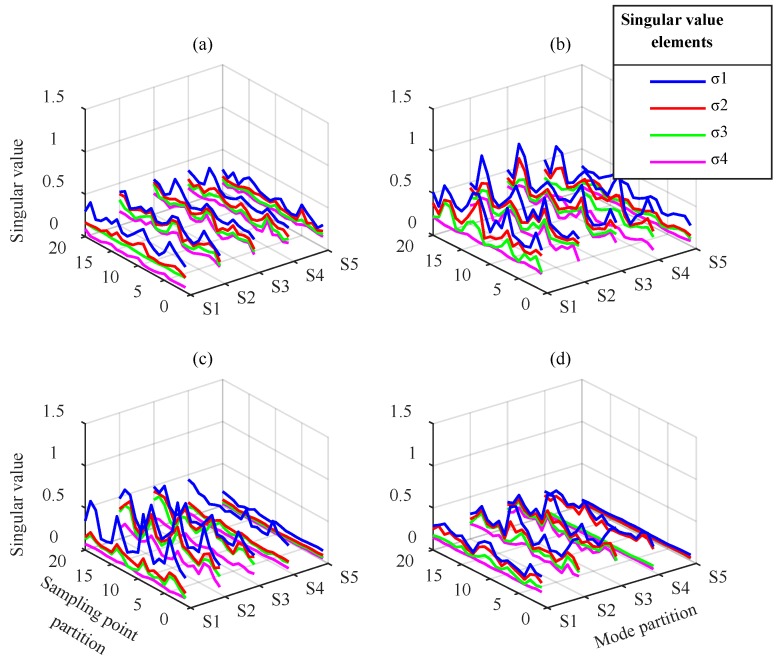
Singular value vector matrices from VMD: (**a**) normal gear, (**b**) gear with wear, (**c**) gear with breakage, and (**d**) gear with root crack tooth.

**Figure 10:**
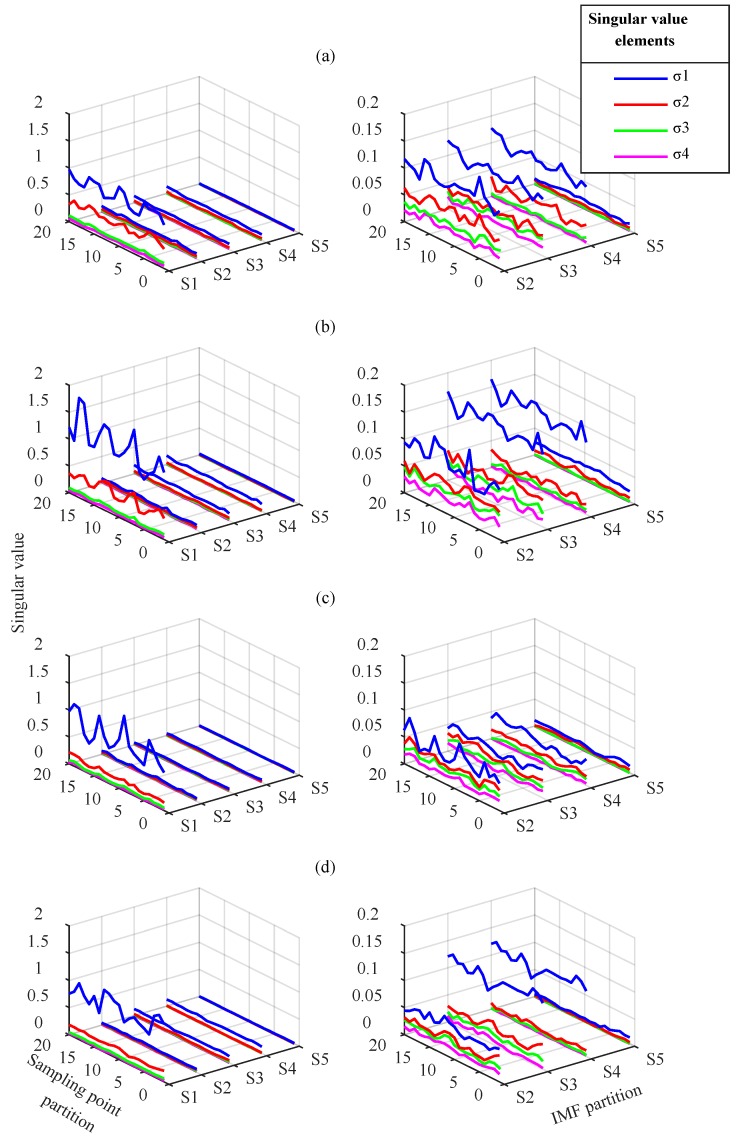
Singular value vector matrices from EEMD: (**a**) normal gear, (**b**) gear with wear, (**c**) gear with breakage, and (**d**) gear with root crack tooth.

VMD modes and IMFs were processed according to the partition extraction method described in Section 2. To extract feature information comprehensively, 4 × 512 submatrices were used to partition the mode matrix. The extraction step length of the modes and the sampling points were 4 and 384, respectively. Using SVD, the singular value vector of each submatrix containing four singular value elements, $\sigma_{1}-\sigma_{4}$, were obtained to construct the 20 × 20 singular value vector matrix of the current fault state. The VMD modes and EEMD IMFs partition sequence corresponding to five groups of singular value vectors named S1–S5 are displayed in Table 2 and the singular value vector matrices are displayed in Figure 9 and Figure 10.

Figure 9 shows the singular value vector matrix obtained by VMD-based partition extraction method. For different gear faults, the matrix elements differences exist at any position in time and mode direction. The waveform of the maximum element of each singular value vector represents the most obvious difference, especially for gear with wear and gear with breakage. This means that VMD performs effective component separation in different frequency bands, and SVD is effective at extracting submatrix characteristics. The fault information is fully extracted, which cannot be distinguished from the simple time-frequency analysis above.

Figure 10 indicates that the elements in singular value vector matrices from EEMD change in different groups. In each single gear state, S1 is obtained from IMF1–IMF4, which mainly contain high-frequency components. The singular values $\sigma_{1}$ and $\sigma_{2}$ in S1 are typically greater than in S2–S5. The singular values and their arrangement forms in S1–S5 have differences in different fault states. These differences are mainly reflected in the largest element $\sigma_{1}$ in S1–S4, whereas other elements are smaller. In general, the feature extraction effect based on EEMD is worse than VMD. Moreover, the modal aliasing of EEMD in mid- and low-frequency bands prevents the effective separation of the signal components. This leads to the low singular value characteristics of the corresponding submatrix, which must be magnified to distinguish the difference. This prevents effective fault classification.

From the above analysis, the distribution and waveform of the submatrices singular values under different fault states are shown to be clearly different. The different information for the different fault states is primarily contained in the maximum singular value of each submatrix of the IMF matrix. Although the latter three singular values are relatively small, they may contain weak fault feature information. For this reason, they are retained in the diagnosis process. Therefore, the singular value vector matrix obtained by partition can be used as the feature parameter to reflect the fault feature information. It can be input to the CNN to achieve fault classification.

Traditional neural networks are typically used to input and distinguish one-dimensional vector data. Before using them to address matrix data, the matrix must be restructured to a vector. Although the reconstructed vector can exhibit the differences in fault states, it can destroy the fluctuation trend relative position in relation to the matrix elements. Using CNN to recognize and distinguish fault feature parameters, the matrix data can be input directly. Because of its special network structure, CNN has more advantages than other neural networks in addressing this type of complex data.

A well-established network structure facilitates the achievement of quality network performance. Although more layers can further abstract the characteristics of the input data, the network structure becomes complex and inefficient. Furthermore, the convolution and pooling of CNN are essentially a kind of dimension reduction process. In this process, the resolution of the data is continuously decreased and details are lost. Therefore, the appropriate number of network layers should consider both the calculation speed and recognition rate; the size of the convolution kernel and pooling region should not be overly large. In this paper, the size of the singular value vector matrix was 20 × 20. The structure of the CNN was built and the network parameters were set accordingly, as indicated in Figure 1 and Table 3, respectively. The established CNN consists of alternately arranging two convolution layers and two down sampling layers. The learning rate is one, the number of training samples is five, and the number of iterations is 100.

Using the singular value vector matrix as the fault feature parameter, 300 random samples were constructed using the original vibration signals of four gear states, including 200 training samples and 100 testing samples. They were used as inputs to train and test the network recognition performance. At the end of the neural network, a feature vector with 48 elements was obtained in feature vector layer F5. It was classified by CNN and finally connected to a vector, whose length was four, as the output of the network. The output vector corresponds to the four states of the planetary gear. After network training, the fault identification ability of the CNN was validated; the results are presented in Table 4. From the test results in Table 4, CNN achieved acceptable results for both VMD and EEMD. The trained network accurately identified and diagnosed different fault states of the second stage sun gear. The recognition rate was 100% for the normal gear, gear with breakage, and gear with root crack tooth. The only two errors appeared in the wear fault diagnosis using the EEMD-based partition method, with a 92% recognition rate, whereas the VMD-based method had a total recognition rate of 100%. The errors could be a result of multiple factors such as the degree of the gear fault, repeated tests of the normal gear, and interference due to environmental noise. 

**Figure 11:**
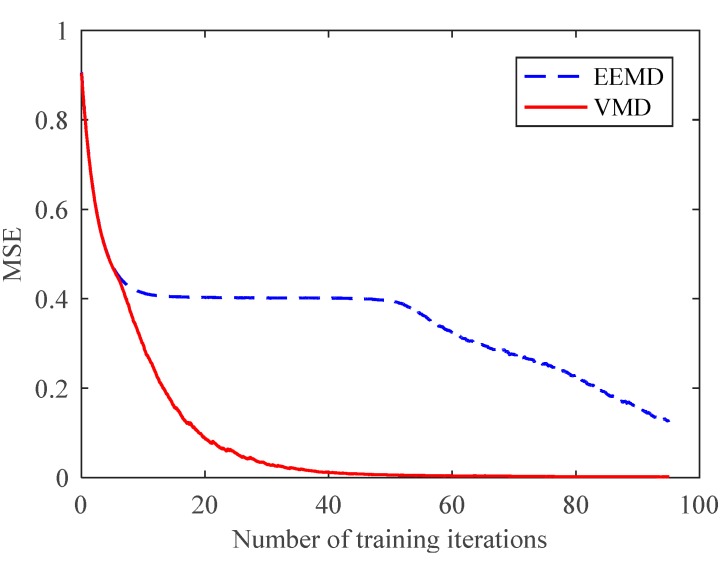
Mean square error (MSE) curves of CNN.

However, further comparative analysis showed the speed advantage of the VMD-based partition extraction method. As shown in Figure 11, the mean square error (MSE) curves of CNN decreased with training time. The MSE of the CNN trained with VMD samples had a much faster decreasing speed, which means a better training effect. In fact, samples from VMD only required 14 iterations to obtain a 100% total recognition rate. This effect has never been achieved by samples from EEMD with training 100 times. The slow training speed of the CNN proves that the EEMD-based partition method is insufficient for fault feature extraction and state classification. In contrast, the advantages of the VMD-based partition method in feature extraction, training speed, and recognition rate are reflected. Based on the above results, the proposed method was proven to effectively extract fault feature information and accurately diagnose planetary gear faults.

## 5. Application in Degradation Recognition of Planetary Gears

To further validate the effectiveness of the proposed method and extend its application field, a breakage degradation experiment of the second stage sun gear was performed and analysed.

**Figure 12:**
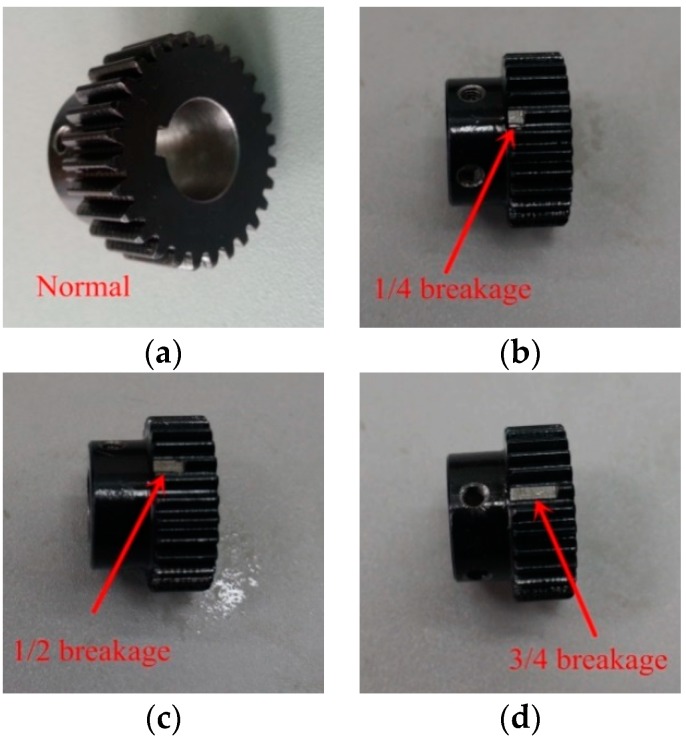
Four breakage degradation states: (**a**) normal gear, (**b**) one-quarter breakage, (**c**) one-half breakage, and (**d**) three-quarters breakage.

**Figure 13:**
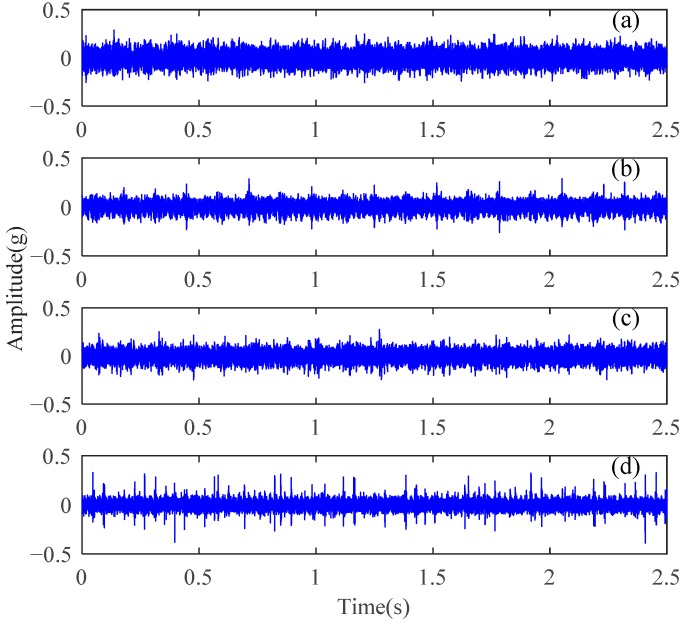
Time-domain vibration signals of breakage degradation: (**a**) normal gear, (**b**) one-quarter breakage, (**c**) one-half breakage, and (**d**) three-quarters breakage.

As shown in Figure 12, the second-stage sun gear degradation experiment was completed using normal gear, gear with one-quarter, one-half, and three-quarters breakage. The motor output frequency was 45 Hz and the other parameters were set the same as in Section 3 and Section 4. Vibration signals were collected and are shown in Figure 13. The differences in the time-domain vibration signals in different degradation states were weaker than in different fault states. This increases the difficulty of feature extraction and recognition.

**Figure 14:**
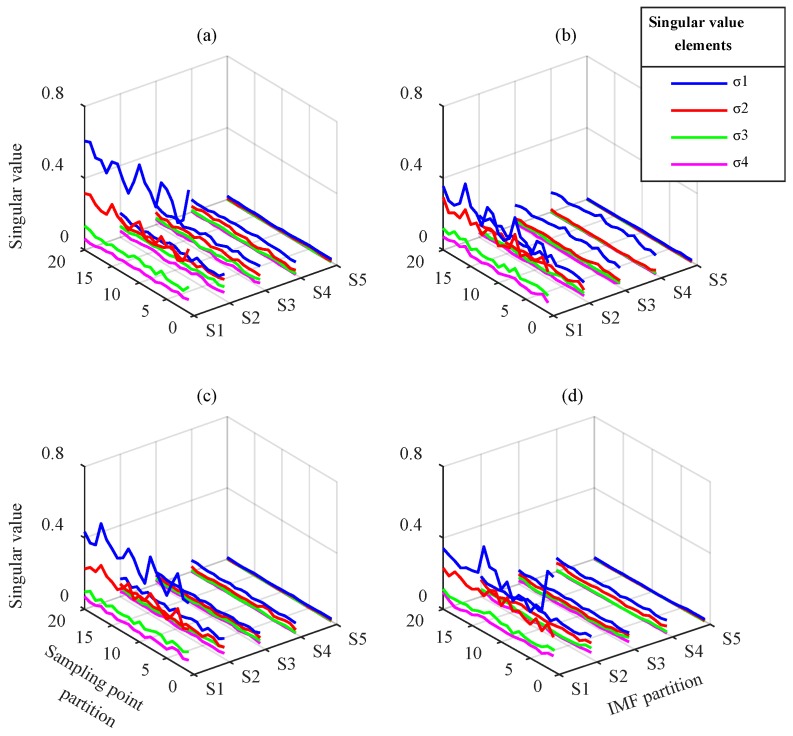
Degradation feature matrices from EEMD: (**a**) normal gear, (**b**) one-quarter breakage, (**c**) one-half breakage, and (**d**) three-quarters breakage.

**Figure 15:**
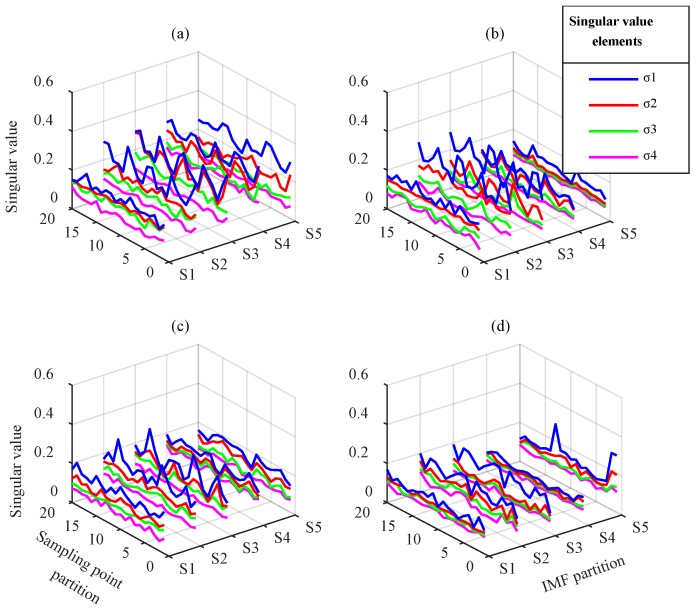
Degradation feature matrices from VMD: (**a**) normal gear, (**b**) one-quarter breakage, (**c**) one-half breakage, and (**d**) three-quarters breakage.

The degradation feature matrices obtained by EEMD and VMD are shown in Figure 14 and Figure 15, respectively. EEMD resulted in poor extraction effect and could not effectively distinguish weak differences in different degradation states. Conversely, in the degradation feature matrices obtained by VMD, all singular value vectors changed with gear degradation. This indicates that the degradation information contained in different frequency bands was extracted synthetically.

**Figure 16:**
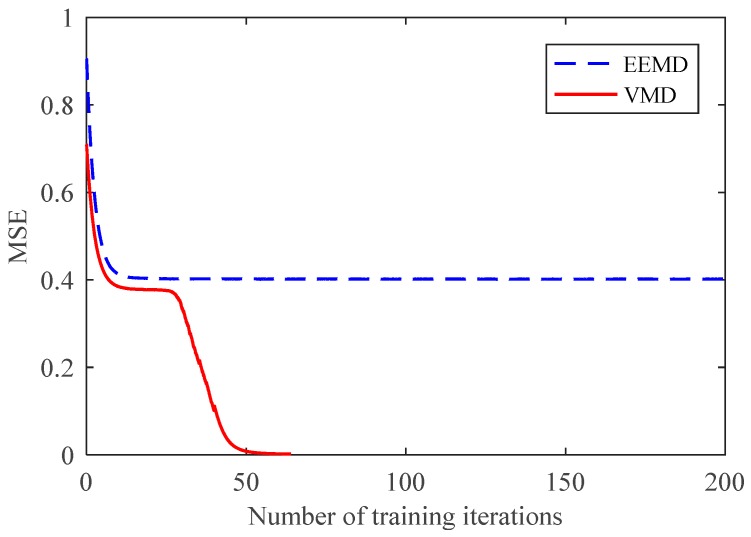
CNN MSE curves of breakage degradation.

A total of 200 training samples and 100 test samples were randomly constructed to input to the CNN. The comparison of the test results is shown in Figure 16 and Table 5. The EEMD-based method had a very low recognition rate and its MSE could not continue to decline, which means that the performance of the EEMD method was insufficient for degradation states with similar features. The MSE curve of the VMD-based method was reduced to nearby 0 after training 70 times, and the recognition rate was 100%. This proves that the proposed method can fully extract feature information and utilize the recognition ability of CNN. It can not only diagnose different faults of planetary gears, but also accurately identify the degradation states.

Notably, the proposed method is just an option for feature extraction and diagnosis. As it can retain the signal information in both the time domain and frequency domains, the method capability under more complex conditions still has room for further research and improvement.

## 6. Conclusions

A novel feature extraction and fault diagnosis method for planetary gears was proposed. The vibration signal of a planetary gear transmission has more intense noise, nonlinear, and nonstationary characteristics because of the poor working conditions and complex transmission structure. These factors cause fault feature information to be weak and easily lost when using traditional feature extraction methods. In the proposed approach, a mode matrix without modal aliasing was obtained using VMD, which was then partitioned into many submatrices. The singular value vectors of the submatrices were obtained by SVD and the singular value vector matrix corresponding to the current fault state was constructed. After analyzing, we determined that the singular value vector matrices of the different fault states have clear differences in element size and waveform. The singular value vector matrices can reflect the planetary gear fault state as a whole. In this paper, the sun gear faults of the second-stage planetary gear were chosen to be studied. The singular value vector matrices from both VMD and EEMD were used as the samples to train and test the CNN, and their results were compared. In the case of 200 training samples, 100 test samples and 100 training times, the total recognition rate of the EEMD-based CNN was 98%, whereas the VMD-based CNN required only 14 training times to obtain 100% total recognition rate. The experimental results confirmed that the proposed feature extraction and fault diagnosis method of planetary gears can successfully extract the weak feature information of gears in different states from the original vibration signals that contain strong interference information and can accurately identify and diagnose planetary gear faults. Further analysis showed that VMD can effectively decompose all frequency components of the signal, which is helpful to the partition extraction method for adequately extracting fault information. In addition, the proposed method showed the potential for use in gear degradation state recognition. Thus, the proposed method is an effective technology for weak fault feature information extraction and diagnosis of planetary gears.

## Figures and Tables

**Table 1:** Basic parameters of two-stage planetary gears.

	First Stage			Second Stage		
	Sun Gear	Planet Gear	Ring Gear	Sun Gear	Planet Gear	Ring Gear
Number of teeth	20	40	100	28	36	100
Fault feature frequency (Hz)	100	16.67	20	20.83	4.05	5.83
Meshing frequency (Hz)	666.67			145.83		

**Table 2:** The partition of modes using variable mode decomposition (VMD) and intrinsic mode functions (IMFs) using ensemble empirical mode decomposition (EEMD).

Singular Value Vector Group	S1	S2	S3	S4	S5
VMD	u1–u4	u3–u6	u5–u8	u7–u10	u9–u12
EEMD	IMF1–IMF4	IMF3–IMF6	IMF5–IMF8	IMF7–IMF10	IMF9–IMF12

**Table 3:** Basic parameters of the convolutional neural network (CNN).

Layer	C1	D2	C3	D4
Feature matrix number	6	6	12	12
Feature matrix size	16 × 16	8 × 8	4 × 4	2 × 2
Kernel size	5 × 5	2 × 2	5 × 5	2 × 2

**Table 4:** Recognition rate of different gear faults.

	Total Recognition Rate	Normal Gear	Wear Fault	Tooth Breakage Fault	Root Crack Fault
VMD	100%	100%	100%	100%	100%
EEMD	98%	100%	92%	100%	100%

**Table 5:** Recognition rate of breakage degradation with training 70 times.

	Total Recognition Rate	Normal Gear	1/4 Breakage	1/2 Breakage	3/4 Breakage
VMD	100%	100%	100%	100%	100%
EEMD	24%	96%	0%	0%	0%

## References

[1] Zhang J., Dhupia J.S., Gajanayake C.J. (2015). Stator current analysis from electrical machines using resonance residual technique to detect faults in planetary gearboxes. IEEE Trans. Ind. Electron..

[2] Feng Z., Chen X., Liang M. (2016). Joint envelope and frequency order spectrum analysis based on iterative generalized demodulation for planetary gearbox fault diagnosis under nonstationary conditions. Mech. Syst. Signal Process..

[3] Chen X., Cheng G., Li H., Zhang M. (2016). Diagnosing planetary gear faults using the fuzzy entropy of LMD and ANFIS. J. Mech. Sci. Technol..

[4] Park J., Ha J.M., Oh H., Youn B.D., Choi J.H., Kim N.H. (2016). Model-based fault diagnosis of a planetary gear: A novel approach using transmission error. IEEE Trans. Reliab..

[5] Cheng G., Cheng Y.L., Shen L.H., Qiu J.B., Zhang S. (2013). Gear fault identification based on Hilbert–Huang transform and SOM neural network. Measurement.

[6] Singh D.S., Zhao Q. (2016). Pseudo-fault signal assisted EMD for fault detection and isolation in rotating machines. Mech. Syst. Signal Process..

[7] Chen X.H., Cheng G., Shan X.L., Hu X., Guo Q., Liu H.G. (2015). Research of weak fault feature information extraction of planetary gear based on ensemble empirical mode decomposition and adaptive stochastic resonance. Measurement.

[8] Xu Y., Luo M., Li T., Song G. (2017). ECG Signal de-noising and baseline wander correction based on CEEMDAN and wavelet threshold. Sensors.

[9] Zhang C., Li Z., Hu C., Chen S., Wang J., Zhang X. (2017). An optimized ensemble local mean decomposition method for fault detection of mechanical components. Meas. Sci. Technol..

[10] Dragomiretskiy K., Zosso D. (2014). Variational mode decomposition. IEEE Trans. Signal Process..

[11] Yan J., Hong H., Zhao H., Li Y., Gu C., Zhu X. (2016). Through-wall multiple targets vital signs tracking based on VMD algorithm. Sensors.

[12] Liu W., Cao S., Wang Z., Kong X., Chen Y. (2017). Spectral decomposition for hydrocarbon detection based on VMD and Teager-Kaiser energy. IEEE Geosci. Remote Sens. Lett..

[13] Jiao J., Zhao M., Lin J., Liang K. (2018). Hierarchical discriminating sparse coding for weak fault feature extraction of rolling bearings. Reliab. Eng. Syst. Saf..

[14] Peng Z.K., Chu F.L. (2004). Application of the wavelet transform in machine condition monitoring and fault diagnostics: A review with bibliography. Mech. Syst. Signal Process..

[15] Caesarendra W., Tjahjowidodo T. (2017). A review of feature extraction methods in vibration-based condition monitoring and its application for degradation trend estimation of low-speed slew bearing. Machines.

[16] Velazquez A., Swartz R.A. (2015). Output-only cyclo-stationary linear-parameter time-varying stochastic subspace identification method for rotating machinery and spinning structures. J. Sound Vib..

[17] Cong F., Zhong W., Tong S., Tang N., Chen J. (2015). Research of singular value decomposition based on slip matrix for rolling bearing fault diagnosis. J. Sound Vib..

[18] Zhang H., Chen X., Du Z., Yang B. (2017). Sparsity-aware tight frame learning with adaptive subspace recognition for multiple fault diagnosis. Mech. Syst. Signal Process..

[19] Feng Z., Liang M. (2016). Complex signal analysis for planetary gearbox fault diagnosis via shift invariant dictionary learning. Measurement.

[20] Golafshan R., Sanliturk K.Y. (2016). SVD and Hankel matrix based de-noising approach for ball bearing fault detection and its assessment using artificial faults. Mech. Syst. Signal Process..

[21] Jiang H., Chen J., Dong G., Liu T., Chen G. (2015). Study on Hankel matrix-based SVD and its application in rolling element bearing fault diagnosis. Mech. Syst. Signal Process..

[22] Tabrizi A.A., Garibaldi L., Fasana A., Marchesiello S. (2016). Automatic damage identification of roller bearings and effects of sifting stop criterion of IMFs. Measurement.

[23] Glowacz A. (2018). Acoustic based fault diagnosis of three-phase induction motor. Appl. Acoust..

[24] Dabrowski D. (2016). Condition monitoring of planetary gearbox by hardware implementation of artificial neural networks. Measurement.

[25] Cheng G., Chen X., Li H., Li P., Liu H. (2016). Study on planetary gear fault diagnosis based on entropy feature fusion of ensemble empirical mode decomposition. Measurement.

[26] Chen Y., Jiang H., Li C., Jia X., Ghamisi P. (2016). Deep Feature Extraction and Classification of Hyperspectral Images Based on Convolutional Neural Networks. IEEE Trans. Geosci. Remote Sens..

[27] Huang Y., Wu R., Sun Y., Wang W., Ding X. (2015). Vehicle logo recognition system based on convolutional neural networks with a pretraining strategy. IEEE Trans. Intell. Transp. Syst..

[28] Chen T., Lin L., Liu L., Luo X., Li X. (2016). DISC: Deep image saliency computing via progressive representation learning. IEEE Trans. Neural Netw. Learn. Syst..

[29] Levine S., Finn C., Darrell T., Abbeel P. (2015). End-to-end training of deep visuomotor policies. J. Mach. Learn. Res..

[30] Silver D., Huang A., Maddison C.J., Guez A., Sifre L., Van Den Driessche G., Schrittwieser J., Antonoglou I., Panneershelvam V., Lanctot M. (2016). Mastering the game of Go with deep neural networks and tree search. Nature.

